# Real-Time 3D Tracking of Laparoscopy Training Instruments for Assessment and Feedback

**DOI:** 10.3389/frobt.2021.751741

**Published:** 2021-11-04

**Authors:** Benjamin Gautier, Harun Tugal, Benjie Tang, Ghulam Nabi, Mustafa Suphi Erden

**Affiliations:** ^1^ Heriot-Watt University, Scotland, United Kingdom; ^2^ University of Dundee and Ninewells Hospital, Dundee, United Kingdom

**Keywords:** real-time motion tracking, cartesian position estimation, single view camera, skill metric, laparacospy, laparoscopy training

## Abstract

Assessment of minimally invasive surgical skills is a non-trivial task, usually requiring the presence and time of expert observers, including subjectivity and requiring special and expensive equipment and software. Although there are virtual simulators that provide self-assessment features, they are limited as the trainee loses the immediate feedback from realistic physical interaction. The physical training boxes, on the other hand, preserve the immediate physical feedback, but lack the automated self-assessment facilities. This study develops an algorithm for real-time tracking of laparoscopy instruments in the video cues of a standard physical laparoscopy training box with a single fisheye camera. The developed visual tracking algorithm recovers the 3D positions of the laparoscopic instrument tips, to which simple colored tapes (markers) are attached. With such system, the extracted instrument trajectories can be digitally processed, and automated self-assessment feedback can be provided. In this way, both the physical interaction feedback would be preserved and the need for the observance of an expert would be overcome. Real-time instrument tracking with a suitable assessment criterion would constitute a significant step towards provision of real-time (immediate) feedback to correct trainee actions and show them how the action should be performed. This study is a step towards achieving this with a low cost, automated, and widely applicable laparoscopy training and assessment system using a standard physical training box equipped with a fisheye camera.

## Introduction

**FIGURE 1:**
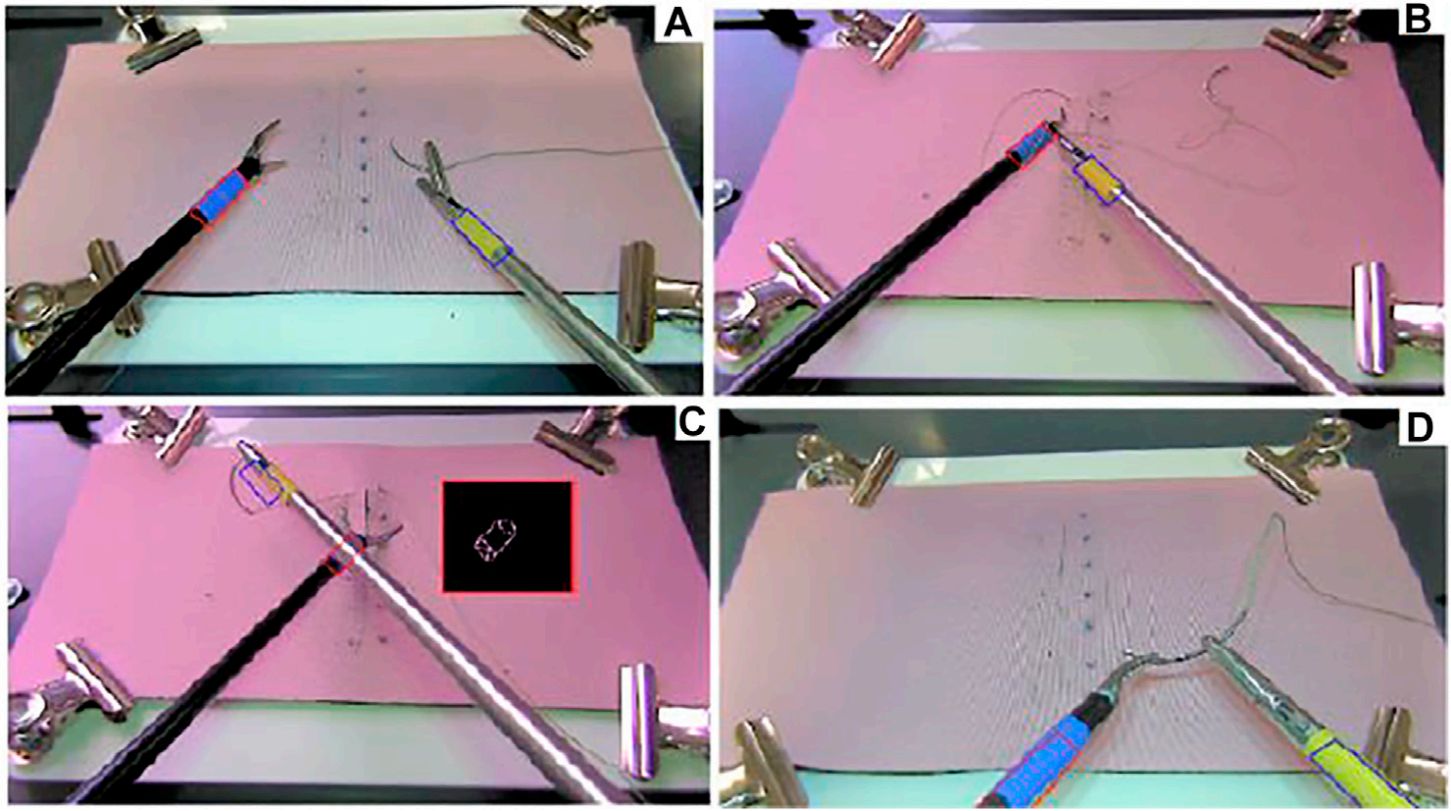
**(A)** Instrument detection, **(B)** convex Hull area when the rope is crossing the blue marker, **(C)** convex Hull area when the instrument is crossing the blue marker and obscuring the marker, **(D)** out of the field of view, when Kalman filter prediction is used (Gautier et al., 2019).

Laparoscopy is a minimal invasive surgery performed in the abdominal cavity with the most important advantage of fast recovery of patients, compared to conventional open surgery procedures. Using only small incisions, the surgeon can perform an operation such as removing parts on organs or retrieving tissue samples for further analysis, without fully opening the abdomen (Eyvazzadeh and Kavic, 2011). However, this method brings new challenges to the surgeon as it is more difficult to perform than a conventional open surgery. The main challenges are a reduced field of view due to the use of a single camera, loss of depth perception, less sensitive force perception, and inverted motions due to a rotation around the insertion point (fulcrum effect) (Xina et al., 2006; Levy and Mobasheri, 2017). Exemplary camera views of a suturing training from the inside of the training box used in this study are seen in Figure 1, as adapted from our previous work (Gautier et al., 2019). To adapt to those challenges a surgeon must carry out an intensive training, which is difficult to be objectively assessed due to the lack of consistent quantitative measures (Chang et al., 2016).

Laparoscopy training aims at motor learning (Dankelman et al., 2005; Halsband and Lange, 2006) for manipulation skills with the laparoscopic instruments. Long training periods and expensive resources are required for training and evaluation of novice surgeons (Gutt et al., 2002; Vassiliou et al., 2005; Berg et al., 2007). Suturing is considered to be one of the procedures that require high degree of manipulations skills in laparoscopy (Judkins et al., 2006; Wang et al., 2010). A major concern in laparoscopy training is about evaluating the degree of skill of surgeons. Mostly offline evaluation techniques are used (Aggarwal et al., 2004; Chmarra et al., 2007), with criteria such as the number of movements of the tool coded by acceleration and deceleration thresholds, the path length covered by the tool-tip, the time taken to bring the tool-tip from one point to another (Datta et al., 2001; Moorthy et al., 2003; Chmarra et al., 2008), and the frequency content of time frames (Megali et al., 2006). These criteria make use of the translational tool-tip trajectory.

For training and assessment of laparoscopy skills there are physical box trainers (Aggarwal et al., 2004; Schreuder et al., 2011; Kunert et al., 2020; Ulrich et al., 2020), visual simulators (Ahlborg et al., 2013; Strandbygaard et al., 2013), and recently also augmented reality systems (Lahanas et al., 2015). The pros and cons of these systems have been discussed in literature (Lahanas et al., 2015). While box trainers provide physically realistic interaction, they require supervision by an expert for training and assessment. Virtual simulator, on the other hand, are limited in physical realism (von Websky et al., 2013; Greco et al., 2010), but allow collection of digital data that can be processed to perform quantified assessment without the need for a supervisor. Augmented reality systems as in (Lahanas et al., 2015) constitute an attempt to bring together the advantages of the two: physical realism of a training box and digital computation of registered data. However, currently such systems (Lahanas et al., 2015) yet come with extra sensors and function with a virtual setup, though with physical instruments.

A promising approach that has been appearing in the recent years is to equip physical training boxes with machine vision and intelligence to assess the physical performance of the trainee (Sánchez-Margallo et al., 2011; Alonso-Silverio et al., 2018). However, the emergent systems such as in (Alonso-Silverio et al., 2018), yet provide assessment/feedback only after the task is completed; in other words, they process the performance offline. With similar spirit, we developed in an earlier study an off-line trajectory tracking algorithm of laparoscopy tool tips in a laparoscopy training box and provided novel assessment methods using the extracted trajectories (Gautier et al., 2019). In the current paper, we present a substantially improved version of our tracking algorithm, which is capable of real-time tracking of the 3D position of instruments with a single camera, and we assess the real-time tracking performance with a Robotic Surgery Trainer setup. Our motivation is that the real-time extracted instrument trajectories can be digitally processed, and automated self-assessment feedback can be provided to the trainee in real-time. In this way both the physical interaction feedback would be preserved and the need for the observance of an expert would be overcome by provision of instant feedback when the trainee makes a mistake or deviates from the optimal way of performing the task.

In this study, we apply our algorithm to the videos of training sessions for intra-corporeal wound suturing, which is considered to be one of the most difficult procedures in laparoscopy training (De Paolis et al., 2014; Hudgens and Pasic, 2015; Chang et al., 2016). For that purpose, we have recorded videos from six professional surgeons and ten novice subjects. Ethical approval was acquired from the Ethics Committee of School of Engineering and Physical Sciences at Heriot-Watt University with Ethics Approval number 18/EA/MSE/1 and all participants provided their Informed Consent prior to data collection. Our real-time tracking algorithm in this paper is successful to extract the same trajectories from these recorded videos as the off-line algorithm we presented in our previous work (Gautier et al., 2019). Therefore, the conventional assessment criteria we used in (Gautier et al., 2019) from the literature (Kroeze et al., 2009; Ahmmad et al., 2011; Retrosi et al., 2015; Chang et al., 2016; Estrada et al., 2016) and the novel one we proposed in (Gautier et al., 2019) are all applicable also to the real-time extracted trajectories in the current study, highlighting the usefulness of the trajectories to distinguish between novice and professional performances. We do not repeat the explanation and application of these criteria in this paper and refer the reader to (Gautier et al., 2019).

Tracking methods for objects in known environments are well known in the literature and have already been used in several studies on laparoscopy, such as 2D tooltip location tracking in laparoscopy training videos for eye-hand coordination analysis (Jiang et al., 2014), 3D laparoscopy instrument detection using the vanishing point of the edges of the instrument’s image (Allen et al., 2011), stereo-imaging with two webcams and markers (Pérez-Escamirosa et al., 2018), monochrome image processing (Zhang and Payandeh, 2002), making use of the position of the insertion points of the instruments (Doignon et al., 2006; Voros et al., 2006), optical flow information in video frames (Sánchez-Margallo et al., 2011), and instrument tracking for calibration purposes for robotic surgery (Zhang et al., 2020). Among these, the ones that target training mostly use colored markers on the tips of the instruments to be tracked. This is justified for training setups as it is easily applicable to any training laparoscopy instrument and it does not impact the performance of the subject. However, a major challenge with marker-based instrument tracking is that the markers might be obscured or they might get out of the field of camera view (Lin et al., 2016), as illustrated in Figure 1. In this paper we also develop a marker based tracking system; but in comparison to the other methods, 1) we address the problem of occlusion and disappearance from the scene by adopting a Kalman filter to estimate the position only in such instances of disappearance from the scene, and 2) we do the tracking for 3D positioning of two instrument tips in real-time with a speed of 25 frames per second by using the geometric features of the markers. We achieve real-time tracking purely based on a single camera image processing from a standard laparoscopy training box. As our system does not add any extra equipment to a standard laparoscopy training box, we consider it to be low-cost and widely applicable as it can easily be applied to any training box.

The rest of the paper is organized as follows. Our implementation of the real-time tracking in 2D images using color-based markers is presented in *Marker Corner Detection in 2D Images*. In *Tool Tip Position Tracking In 3D*, we explain the method used for 3D Cartesian position estimation in real-time. In *Testing And Verification*, we compare the performance of trajectory extraction with respect to the ground truth trajectories generated by a Robotic Surgery Trainer setup incorporating two UR3 universal Robots. *Conclusion* concludes the paper.

## Marker Corner Detection in 2D Images

**FIGURE 2:**
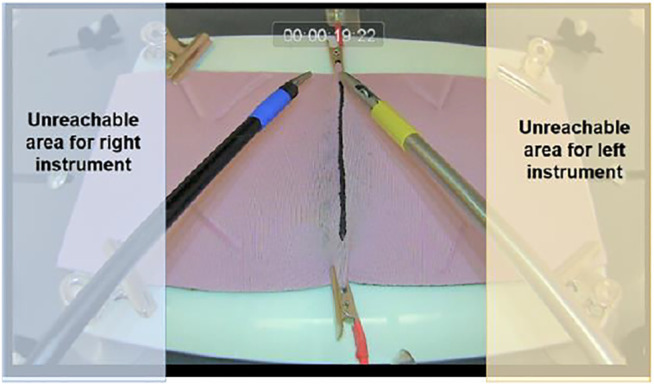
Practical workspace for both instruments based on the collected data.

Instrument detection is realized by tracking the colored tapes attached to the end of the two instruments as in Figure 1 and Figure 2. The colors of the tapes are chosen to be easily separable from the background (usually a pink colored suturing pad) and each other in a Hue Saturation Value (HSV) space. The tracking problem in this setting can be described as subject to a close-to-invariant light exposure (closed environment and short time recording). The image processing techniques used in this study are individually well known in literature; therefore, we will only mention them briefly without detailed explanations. We note that, what we have performed in this study is adapting these techniques and integrating them effectively to solve the specific detection challenges in the context of a laparoscopy training practice. For example, and specifically, the method we have developed allows detection of the corners of a markers even when some parts of the marker are separated from each other in the image, which can happen in two different cases: when the rope is wrapped around the instrument over the tracked marker as in Figure 1B, and when one of the instruments obscures part of the tracked marker on the other instrument as in Figure 1C. The real-time tracking process is realized using a two steps method, a *detection* step where the four corners of a colored tape on each instrument are found in the current 2D image frame (explained in the following sub-sections) and a *tracking* step where a 3D position of the instrument tips are generated using the detected corners (explained in *TOOL TIP POSITION TRACKING IN 3D*).

### Preprocessing

Using the recorded video retrieved from our experiments, an HSV color database is constructed for the range of the pink background pad and for the range of the colors of the tapes on the instruments, across 20 videos recorded from six professional and 10 novice subjects. The HSV range for the tapes is identified by isolating 50 × 50 square regions containing each tape. The mean HSV values are then extracted and used to create the database to be compared to new inputs (Riaz et al., 2009; Abter and Abdullah, 2017). The current database comprises three different illumination setting across the 20 videos: a natural light recording setup, and two artificial light recording environment, one in our laboratory and one in the medical facilities. When a new pad is used in any lighting condition, its detected color is compared to the dataset using a minimum distance formula, and the closest corresponding HSV range is selected for each instrument for the detection.

The second part of the preprocessing is a full frame detection. Using color space conversion (cvtColor in OpenCV) on the full image is time consuming; therefore, it is performed only on the first two video frames. The marker positions in the image are retrieved and the center of gravity of each detected contour around the marker is used to estimate the position of the contour in the next frame using the motion gradient. Finally, the extrinsic parameters of the camera are retrieved using the perspective transformation matrix based on the pink pad background corners in the very first frame. Then the Euler angles representing the camera orientation are extracted, as the camera height being adjustable and hence might change across the use of the system in different times. In our setup this initialization is applied automatically every time the system is turned on.

**FIGURE 3:**
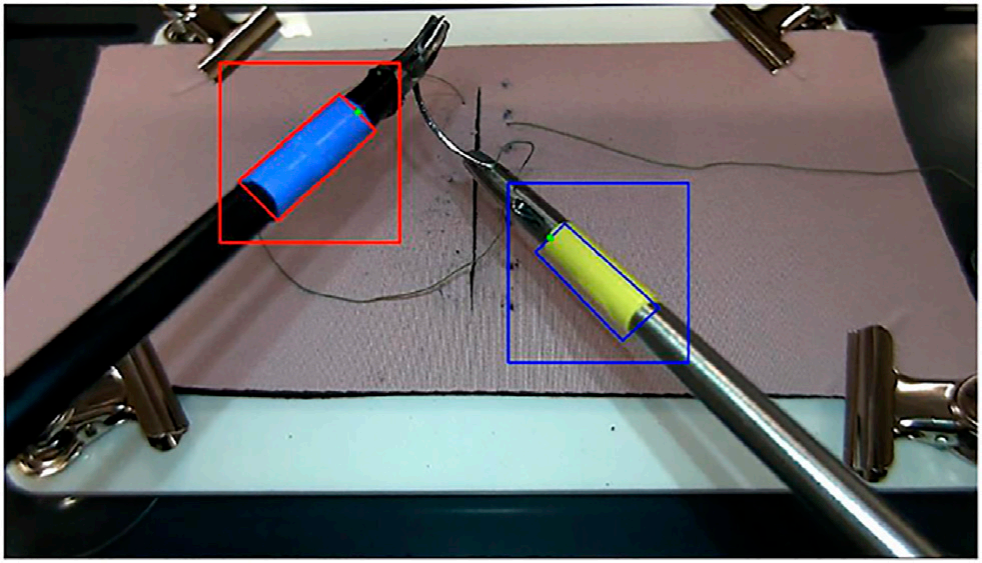
Sample ROI squares around the markers.

Our detection process follows a general framework for HSV object detection (Cucchiara et al., 2001; Hamuda et al., 2017) with a real-time adjustable detection window for time efficiency. The embedded camera has a 25 frames-per-second (fps) reading rate thus the full process needs to be designed to have a minimum of 50-Hz response to generate the position of two instrument tips in each frame cycle. In order to obtain the Cartesian information at a rate of minimum 50 Hz, the detection process is only applied on a windowed section of the frame centered around the estimated position of the instruments. The center of gravity of the marker tapes $\left(c_{g}\right)$ in the next image frame is estimated using the gradient of motion found in the region of interest (ROI) in the previous frames. ROI is defined as a square window where we estimate the marker to be inside in each frame (Figure 3). Using the estimate of the gradient of motion in the previous frame, a new ROI is generated in each frame and the search for the tip position is performed only in this ROI, rather than the whole image. The size of the single edge of the ROI window, ω , which is adjusted in each step, is computed as follows:

$$\omega =\left[a_{\text{prev}}\times \left(\frac{\omega_{\text{max}}-\omega_{\text{min}}}{a_{\text{max}}}\right)+\omega_{\text{min}}\right]\times \left[\frac{\left||\nabla c_{g}|\right|}{c_{g,v\text{max}}}+1\right]$$ (1)

where $a_{\text{prev}}$ is the area of the detected marker in the previous image, $a_{\text{max}}$ is the empirically identified maximum area of a marker in an image when the marker is closest to the camera (18,000 pixels), $\omega_{\text{max}}$ is the maximum edge size of ROI set to 400 pixels, $\omega_{\text{min}}$ is the minimum edge size of ROI set to 100 pixels, and $c_{g,v\max}$ is an empirically chosen value for the maximum speed of the tool tip across frames set to 30 pixels per frame. The factor on the left hand side of the equation handles the size of the window of ROI based on the prior knowledge of the instrument size and the factor on the right hand side adjusts the size of the ROI based on the velocity of the instrument computed on the previous frames. The estimation of the $c_{g}$ and adjustment of the size of the search window (ROI) for each frame are the keys to achieving a fast-enough detection allowing real-time tracking.

In order to apply a region of interest with the dynamic size detection process, some specific cases must be defined and handled properly to avoid wrong detection and thus losing the instruments. The specific cases are identified as follows:

• The area is not consistent with the previous detection.
• The velocity of the instrument is not realistic.
• The detection algorithm could not find a set of four corners in the previous ROI.

If any of these special cases is detected, the next position estimation is rendered incorrect and the ROI cannot be computed, thus a search of the instrument is applied on a larger part of the image. This detection also is not made on the full image. A notion of dead space is identified based on the recorded dataset which leads to identification of a “practical workspace” for the instruments as in Figure 2 and the search is conducted only in this workspace.

**FIGURE 4:**
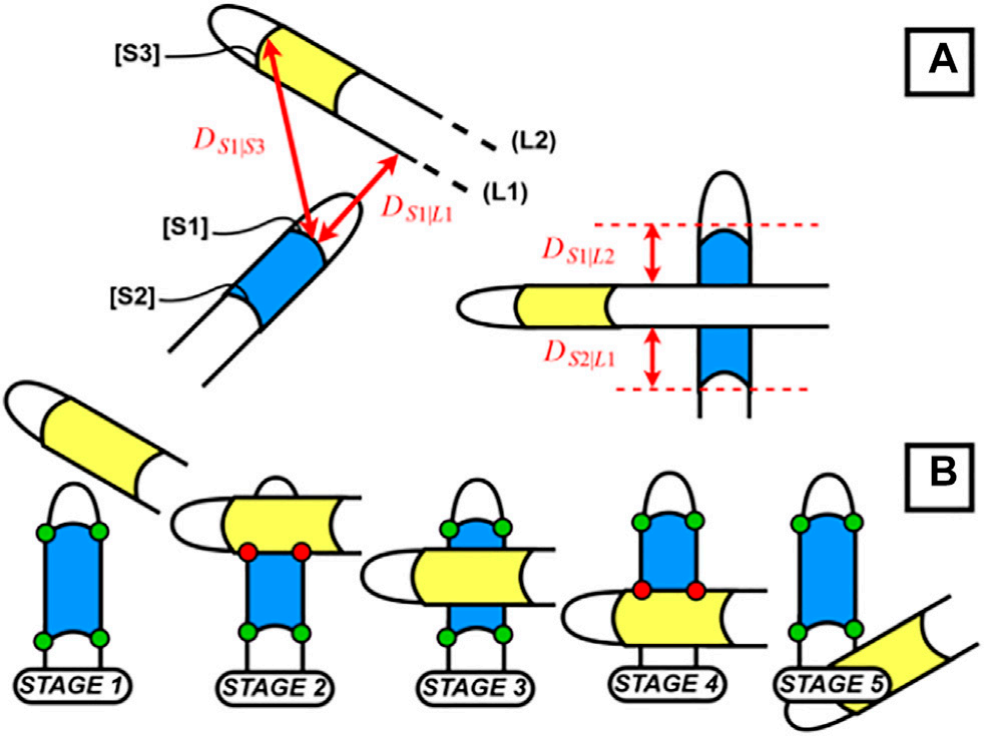
**(A)** Distance between instruments; **(B)** crossing between instruments.

One of the challenges with two instrument detection is the crossing event when one of the instruments is obscured or partially obscured by the other (Figure 1C, Figure 4). To deal with such crossing, the separated parts of the overall contour of the obscured marker are detected using Canny detection and then a convex hull is created using the detected parts. This method allows a regrouping of the separated parts to deal with the situation illustrated in Stage 3 of the crossing (Figure 4B). Furthermore, it allows to simplify the representation of the geometry of the contour and thus speeds up the process of finding the corners using Hough transform.

Another advantage of using a convex hull representation is the following. In order to speed up the detection process, we are directly using the raw (non-flattened) fisheye camera output, thus the shape retrieved before application of the convex hull does not have straight lines. This would result in having a large set of candidate points for corner selection after the Hough transform. This is avoided and the number of the candidate points for the corners is narrowed down by adapting a convex hull. Using the raw feed also results that we cannot directly identify the correct set of four points in the Hough transform output. To overcome this, we flatten the output points from the Hough transform. In this way, we apply flattening only to about 40 points at the end of the detection, instead of approximately 2 million initially in the frame. For flattening, we use the intrinsic parameters of the camera (distortion, focal lengths and focal points) retrieved in advance from a chessboard calibration.

**FIGURE 5:**
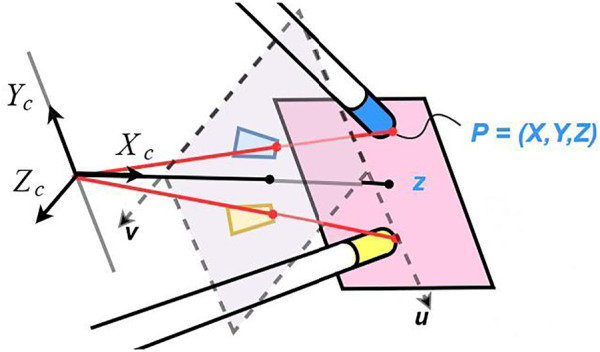
Representation of the trapezoidal shapes after flattening the view of the cylinders and the angle of the instruments regarding the camera.

**FIGURE 6:**
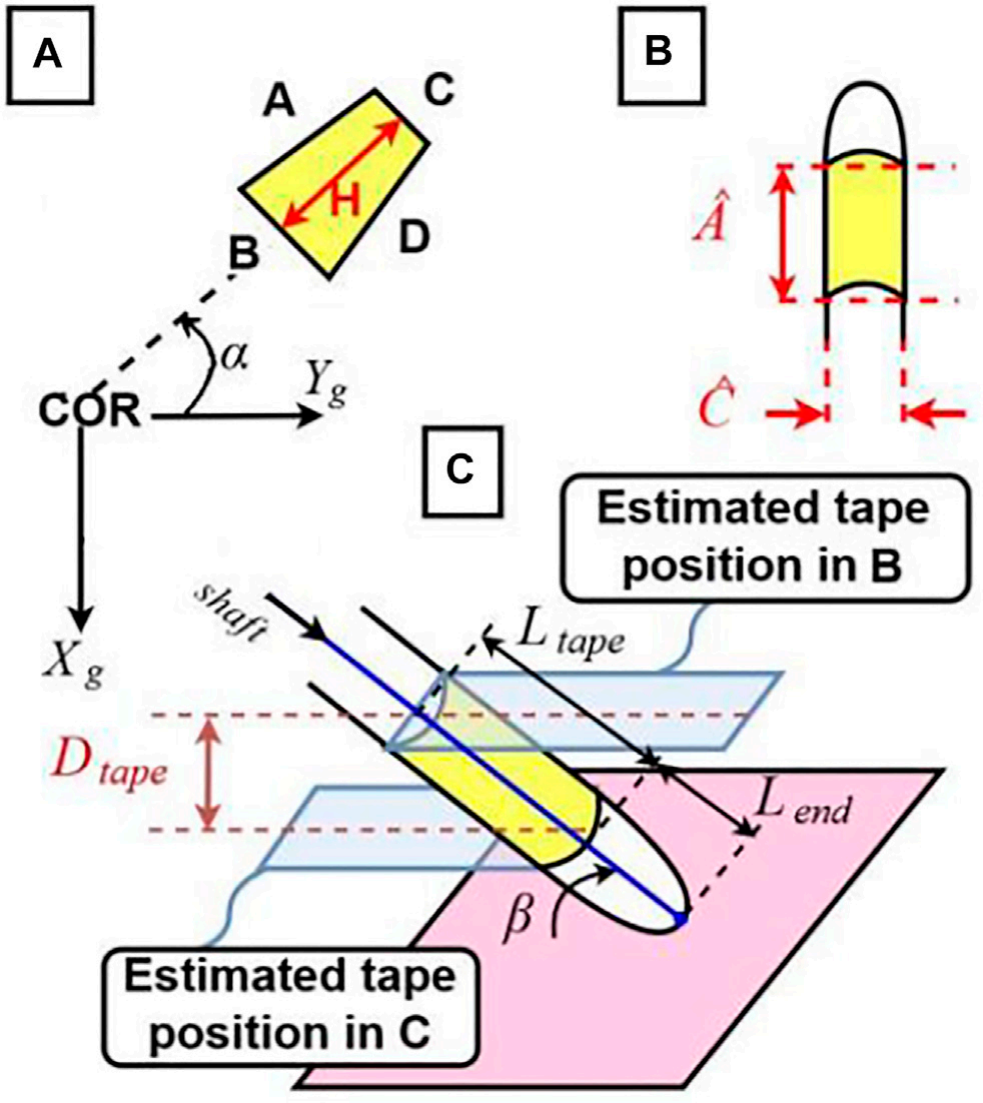
**(A)** Estimated α angle, rotation around Z of the instrument around the Center of Rotation and parameters of the trapezoidal detection; **(B)** parameters of the real instruments; **(C)** estimated β angle, rotation around X.

**FIGURE 7:**
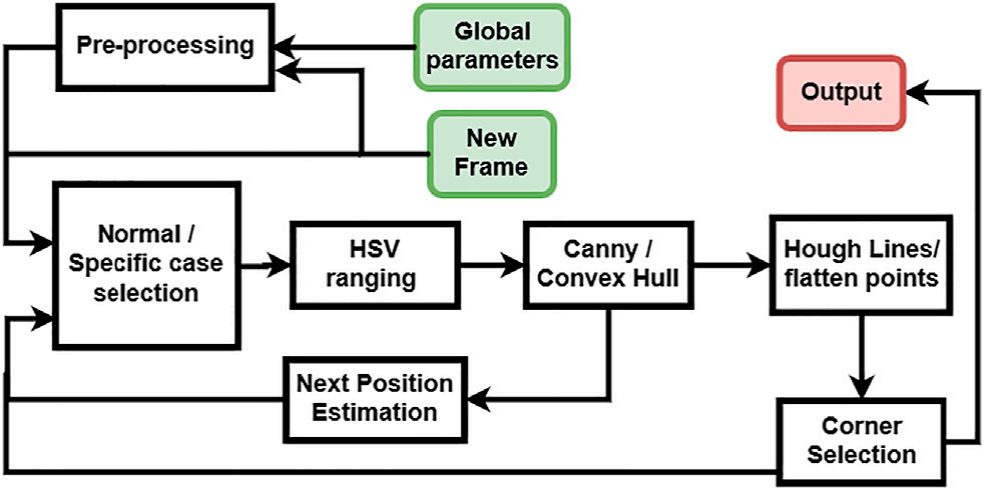
Diagram of the detection process with image processing.

For the corner selection we use the knowledge on the contour pose in the image and the properties of the trapezoidal shape when the cylinders (markers) are viewed from top (Figure 5) where the detected line segments C and B in Figure 6A must remain parallel. This method allows to find the best candidates for the corner points from the list output in the previous steps. The full detection process with image processing can be summarized with the block diagram in Figure 7.

### Estimation for Missing Corners

In the previously mentioned specific cases, a correct corner detection is not possible with the image processing as explained up to this point. In these cases, a Kalman filter is used to estimate the position of the instruments. The Kalman filter implementation is a standard one where the estimation of the next position of the instrument is based on a corrector and predictor equation (Nguyen and Smeulders, 2004; Chen, 2012). The corrector uses the previous measurement to update the model and the predictor estimates the next position using the error covariance of the model. In our application, we use the Kalman filter estimation only when marker corners cannot be found through previously explained image processing procedures. Deciding on the fly when to switch between the actual detection and Kalman filter prediction is not a trivial task. Usually the algorithms that detect occlusions use the geometric properties of the object and therefore are computationally expensive. In this study a different approach is developed using the specificities of the laparoscopy training environment and specifically the distance between the two instruments. The markers can be missing in the laparoscopy training context in two cases, first, some corners being occluded by the crossing of the instruments (Figure 4B) and second, the instrument being outside the field of view (Figure 2D). For the first case, our method relies on the detection of the two instruments being close enough to each other for one of them being occluded by the other. For that purpose, we compute the minimal distance between a detected segment and the line formed by the other segment or the tip of the other instrument, using the following formulas:

$$\text{sign}\left(d_{S_{j}|L_{i}}\right)=\text{sign}\left(\frac{a_{i\;}.x_{m_{j}}-y_{m_{j}}+b_{i\;}}{\sqrt{1+a^{2}}}\right)$$ (2)

$$\text{sign}\left(d_{S_{j}|S_{i}}\right)=\text{sign}\left(\sqrt{{\left(x_{m_{j}}-\;x_{m_{i}}\right)}^{2}+{\left(y_{m_{j}}-y_{m_{i}}\right)}^{2}}\right)$$ (3)

where $a_{i}$ and $b_{i}$ are the parameters of the line $L_{i_{i=1,..,4}}$ and $\left(x_{m_{j}},y_{m_{j}}\right)$ is the middle point of a given segment $s_{j}$ as described in Figure 4A, and d stands for distance The sign of the computed distances between the edge lines in the above formulas can be used to detect the occlusions. When an occlusion is detected as such, the Kalman filter estimation is used. Furthermore, in order to reduce the use of the Kalman filter prediction, it is possible to detect the instances when recovery of the actual corners of the markers is possible during the occlusion of the marker body. In Figure 4B, we can see that during Stage 3, the corners of the markers can be completely recovered.

The second situation that requires the Kalman filter estimation is when one of the instruments goes out of the video frame which can simply be estimated and detected using the minimal distance between the previously detected location of the instrument and the borders of the image frame. For such cases, the Kalman filter estimation is directly used. Finally, the four corners of both instruments estimated by the Kalman Filter are flattened similarly as in the previous sub-section.

### Overall Procedure of Detection of Marker Corners

The following are the overall steps of procedure applied for detection of the marker corners in the 2D images as explained in this section:

1. Check whether any of the “specific cases” applies (such as the detection could not find four corners in the previous ROI); if so, perform Pre-processing in the practical workspace (Figure 2); otherwise continue with Step 2.
2. Identify the new ROI (Eq. 1).
3. Apply HSV decomposition in the ROI.
4. Apply Canny edge detection
5. Apply Convex Hull regrouping to obtain a connected contour representing the marker.
6. Apply Hough transform for line detection.
7. Identify the candidate corners and apply flattening at these corner points.
8. Identify the four corners of the marker using the information of geometric relations.
9. Check if any marker is occluded (Equation 2 and Eq. 3) or out of view; if, yes, use Kalman Filter output to estimate the location of the occluded corners and apply flattening at the estimated corners; otherwise stay with the identified corners in Step 9.
10. Output the corner coordinates for depth estimation.

## Tool Tip Position Tracking in 3D

In order to track the full 3D position of the tip of the instruments, we first reconstruct the 2D position information from the 2D Camera view following the methods explained in *Marker Corner Detection In 2D Images*. Afterwards, we estimate the depth using the difference between the computed circumference of the detected marker polygon as seen in the image and the actual circumference of the polygon.

### Real-Time 3D Tracking

As seen in Figure 2, there are two instrument tips, each with four degrees of freedom actively controlled by the subject. However, in this study we track only the three degrees of freedom, the translational movements of each instrument, and ignore the rotational movement around the shaft axis. This is because, almost all criteria of performance that apply to instrument movements in laparoscopy training (Aggarwal et al., 2004)- (Megali et al., 2006) make use of the 3D position of the instrument tips, but not the orientation of the tip. The tip point trajectories without the orientation provide a rich enough information for assessment purposes in laparoscopy training exercises.

**FIGURE 8:**
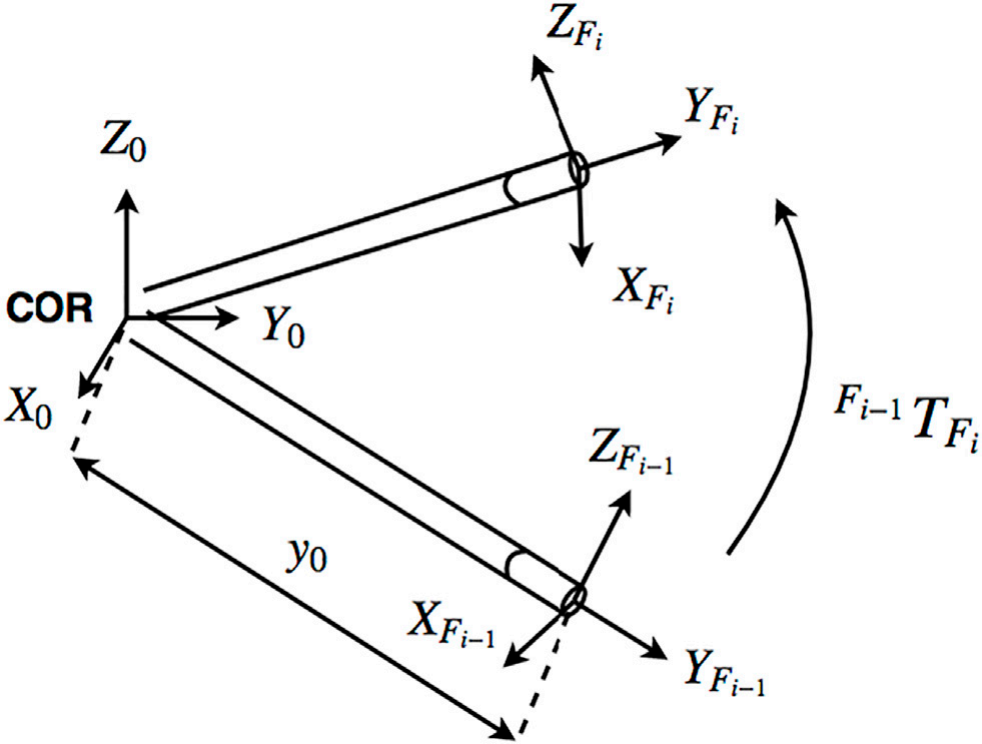
Reference frames at the center of rotation and tool tip (Gautier et al., 2019).

The three degrees of freedom translational movement of the tip point can be represented by (or translated into) other movement parameters, possibly some of them defined as rotations around specific axes, such as rotation of the instrument shaft around an axis through the insertion point. In this study, we consider successive elementary transformations with respect to the “current reference frame” constructed after each transformation (Craig., 2005): specifically, a rotation of the instrument shaft at the insertion point with an angle *α* around the *z* axis of the ground frame, rotation with an angle *β* around the *x* axis of the intermediary frame, and a translation of the tip point along the instrument shaft in *y* axis of the successive intermediary frame, as shown in Figure 6. These three motion parameters can easily be translated into the tip point translation parameters along the three Cartesian axes of a global reference frame through straightforward geometric relations. Let **R**_**0**_ be the orthogonal global reference frame with *x* and *y* axes parallel to the ground and its origin at the instrument center of rotation (COR) (the insertion point) and **R**_**F**_ be the reference frame located at the tip of the instrument ( $R_{F_{i-1}}$ and $R_{F_{i}}$ for each instrument, respectively), as illustrated in Figure 8. A homogeneous transformation matrix, TF0 , can be computed in between the **R**_**0**_ and **R**_**F**_ reference frames in terms of the mentioned elementary rotation and translation matrices as.

TF0=Rot(z,α).Rot(x,β).Trans(y,y0) (4)

to transform the representation of a point in **R**_**0**_ to that in **R**_**F**_ as.

(x0y0z01)=TF0(xFyFzF1). (5)

Tracking the tip point of an instrument corresponds to identifying the α , β , and $y_{0}$ parameters in the above transformation matrix, which can then be used to find the Cartesian position of the tip of the instrument with respect to the global reference frame **R**_**0**_. Our approach is first to identify the α and β angles, using the computed depth difference between the front ( C edge in Figure 6A) and rear ( B edge in Figure 6A) segments of the marker along with the geometric relations as shown in Figure 6. The depth estimation, d , is based on the ratio of the perimeter of the detected marker in the 2D image to the actual perimeter of the marker as in Equation (6),

$$d=\frac{p_{{\text{Tool}}_{\text{|real}}}}{p_{{\text{Tool}}_{\text{|img}}}}$$ (6)

where $p_{{\text{Tool}}_{\text{|real}}}$ represents the actual perimeter of the marker and $p_{{\text{Tool}}_{\text{|img}}}$ represents the computed perimeter from the image. First, we consider the front segment, the edge C in Figure 6A and use this as the image correspondence of the actual length $\hat{C}$ of the marker. We already know the ratio between $\hat{A}$ and $\hat{C}$ segments of the actual ma rker in Figure 6B and using this ratio we can compute a length $A_{C}$ as the image correspondence of the actual edge, ${\hat{A}}_{i}$ . Using these, the perimeter of the rectangle in the image at the location of C can be computed as $2\left(C+A_{C}\right)$ . We can then compute the depth of the rectangle parallel to the ground as located at C (Figure 6C). Following the same procedure, we can also compute the depth of the rectangle parallel to the ground as located at B . The difference between these two depth values provide us with the depth difference due to inclination, $D_{\text{tape}}$ value, indicated in Figure 6C. Knowing the actual width, $\hat{A}$ and the depth difference we can compute the β angle. For α , the angle between the line connecting the centers of C and B edges and the *y* axis of the global reference frame is computed (Figure 6A)*.* Once β is known, the actual length of the instrument can be computed considering the visible length in the image and the inclination angle β , along with the ratio between the actual length and the visible length in the image when the instrument is straightly aligned parallel to the ground (perpendicular to the camera view).

### Testing for Real-Time Processing

In this section we present our analysis of the speed of processing of the overall algorithm in terms of frames-per-second (FPS), with respect to the compression rate we use in streaming the video to the computer and considering the success rate of detection of the corner points of the marker at an instrument-tip.

**FIGURE 9:**
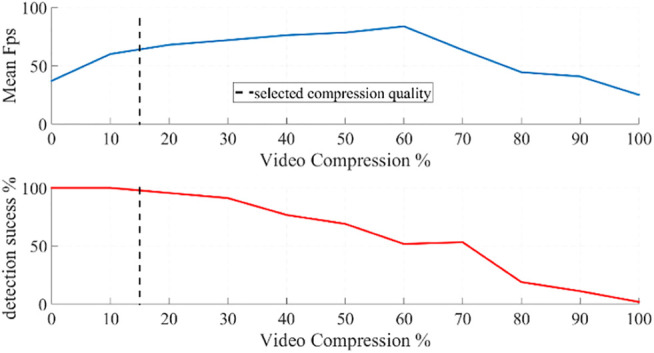
The average speed of image processing to detect the corners of a marker on a single instrument in terms of frames-per-second (upper figure) and the rate of correct detection (lower figure) with respect to varying compression rate of video frames transmitted from the camera to the computer.

In order to achieve a fast processing, we use a streaming communication (TCP/IP) between the camera and the computer housing the image processing software. For that purpose, we apply a compression process on the video feed (on the slave side) to ensure fast and smooth streaming prior to tracking (on the master side). The rate of compression for the streaming is a major factor that impacts the overall speed and performance of detection. We use a JPEG compression and Figure 9 presents the results depicting the speed and performance of detection with varying compression rate. As it is observed in this figure, below 60% compression, the speed of processing increases whereas the performance for correct detection decreases monotonically. In this graph, 15% compression seems to be an optimal choice to achieve a sufficiently fast speed (above 50 Hz) and a high rate of correct detection (very close to 100%); therefore, we applied 15% compression throughout the tests presented in the following section.

## Testing and Verification

**FIGURE 10:**
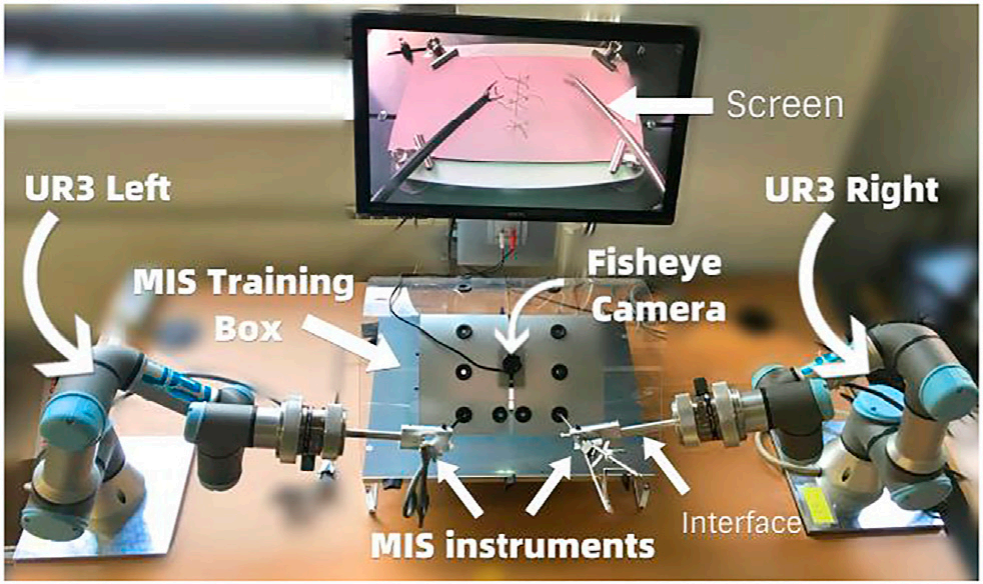
Robotic Surgery Trainer setup used in this study to test the position accuracy of the real-time tracking algorithm.

In our previous study (Gautier et al., 2019) we had tested our off-line tracking algorithm with human subject experiments, where a subject manually manipulated one of the instruments to make its tip to follow the edges of a rectangular object with known dimensions, and where we used the shape and dimensions of the object as a reference for measurement. That method did not distinguish between the actual measurement error of the image processing algorithm and the deviation of the trajectory from the edges of the box due to human hand tremor. Therefore, in the current study we make the measurements with a robotic manipulation setup, the Robotic Surgery Trainer system in our lab incorporating two UR3 universal Robots to manipulate the laparoscopy instruments (Figure 10). With this setup we can accurately record the ground truth positions of the tip of the instruments through the position data provided by the encoders of the robots.

In order to compare the instrument tip trajectory recorded by the robot to that estimated by the real-time tracking algorithm, we first transform the trajectory retrieved from the video tracking into the robot base frame. We then synchronize the two datasets as the robot recording frequency is 125 Hz, giving us a larger number of points in the robot trajectory compared to the tracked trajectory on the video. We eliminate the Euclidian distance between the numeric values of robot recorded and tracked trajectories considering the initial and final points of the trajectories, in order to align them as closely as possible. We then apply a zero-padding in frequency domain to equate the sample size of the position data in the two trajectories. Finally, we apply a norm distance measure between the data of every corresponding couple in the two trajectory data sets to find out the maximal distance between the two trajectories.

**FIGURE 11:**
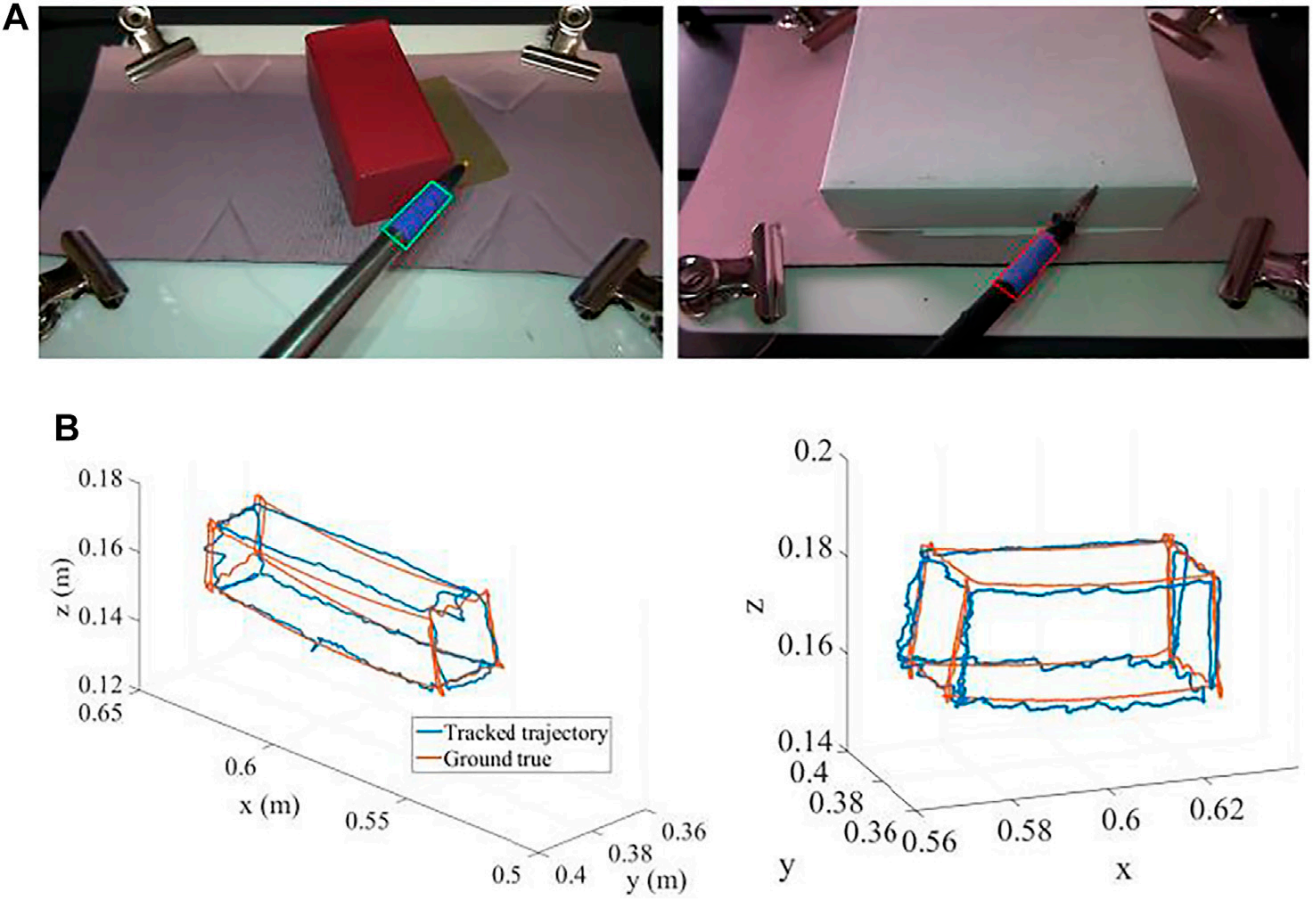
**(A)** Two sample boxes used to generate the trajectories. **(B)** The trajectories generated by the robot (red) and tracked by the real-time image processing algorithm (blue) (units: m).

For this measurement, we again considered the boxes we had used in the previous study (Gautier et al., 2019) (Figure 11A), but this time instead of tracing the actual edges of the physical boxes, we made the robots generate the motions to follow the edges of hypothetical boxes with the tip of the instrument, without the physical presence of the box. In this way all six edges were reachable by the instrument as in Figure 11B. In this figure the red lines show the trajectory followed by the tip of the instrument as recorded by the robot and blue lines show the estimated trajectory as tracked in real-time by the image processing algorithm presented in this paper. We used four different rectangular boxes: a small box occupying half of the screen, a thin box occupying half of the screen, a large box occupying a large space in the screen, and a large and thin box occupying a large space in the screen. Those experiments were realized using both left-hand and right-hand instruments.

The maximum error between the estimated trajectories compared to the robot recorded trajectories through all the experiments was computed to be 1.5 mm in *x*, 4 mm in *y*, and 3 mm in *z* coordinates along the edges of the boxes as in Figure 11B. This performance is sufficient for our purposes to assess skill level with typical criteria as we applied in (Gautier et al., 2019).

## Conclusion

**FIGURE 12:**
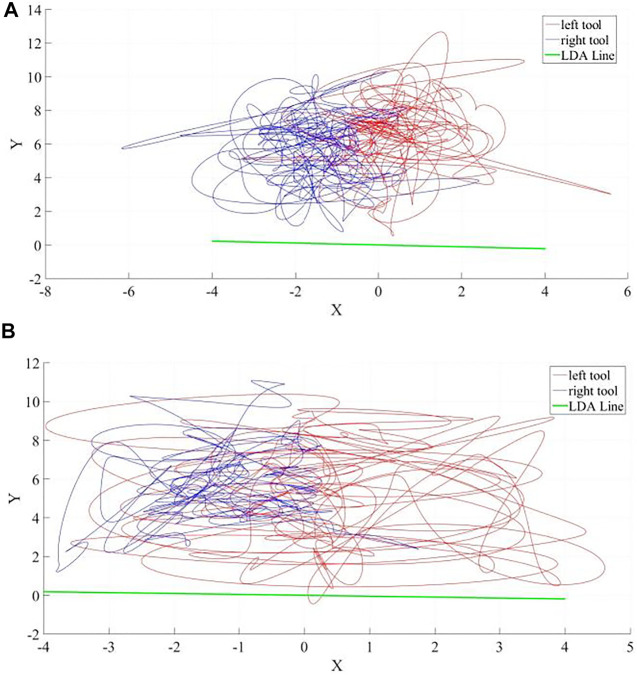
Sample laparoscopy instrument tip-point trajectories (units: cm) as successfully discriminated to belong **(A)** to a professional and **(B)** to a novice by our novel assessment criterion based on Linear Discriminant Analysis (LDA) presented in (Gautier et al., 2019). The right-hand instrument (the driver) trajectory is in red and the left-hand instrument (the receiver) trajectory is in blue. The LDA line in green shows the best direction to distinguish the right-hand and left-hand instruments according to their spatial distribution and as expected it is almost the same in each case in this specific suturing exercise; reflecting that the orientation of the suturing line is the same and perpendicular to the axis that separates right and left hand tools.

In this paper a real time 3D instrument trajectory tracking is developed for single camera laparoscopy training boxes. Trajectories extracted in real-time would be useful to perform real-time skill assessment and to provide real-time feedback, immediately as the subject performs unskilled motions. The work here is a first step towards achieving that goal, as it provides the facility for real-time trajectory extraction. The next step to build on this work would be to develop the assessment criteria that would function in real-time and that would be in such a characteristic to provide immediate feedback to the trainee. The criteria that would serve that purpose are yet to be developed and tested. In our previous work (Gautier et al., 2019), we demonstrated a novel criterion based on the detection of the spatial distribution of the tip positions of the right-hand and left-hand instruments, which functioned significantly superior to existing conventional criteria in literature to distinguish between professional and novice performances we had recorded. The criterion is mainly based on spatial positions of the tips and checks whether the right-hand instrument tip is in its required region in the right-hand side section in the box, and does the same for the left-hand instrument (Figure 12). As this criterion does not rely on history of the positions, we consider it to be promising to be adapted with the presented real-time tracking algorithm to instantly check the performance and generate useful real-time feed-back to the trainee. The real-time tracking algorithm developed in the present study and a potential adaptation of the assessment criterion presented in (Gautier et al., 2019), or similar others yet to be developed, together would be a significant step towards a self-training system with real-time feedback, which would eliminate the need for an expert human trainer, would be low-cost, and would be widely applicable with standard and single camera laparoscopy training boxes. Our future work will progress in this direction.

## Data Availability

The datasets presented in this article are not readily available because the topic of the paper is about image processing to track objects in the videos collected and the content of the videos themselves are not necessary for the topic presented. Requests to access the datasets should be directed to Dr. Mustafa Suphi Erden (m.s.erden@hw.ac.uk).
